# Direct Observation of Structure and Dynamics of Photogenerated Charge Carriers in Poly(3-hexylthiophene) Films by Femtosecond Time-Resolved Near-IR Inverse Raman Spectroscopy

**DOI:** 10.3390/molecules24030431

**Published:** 2019-01-25

**Authors:** Tomohisa Takaya, Ippei Enokida, Yukio Furukawa, Koichi Iwata

**Affiliations:** 1 Department of Chemistry, Faculty of Science, Gakushuin University, 1-5-1 Mejiro, Toshima-ku, Tokyo 171-8588, Japan; 2 Department of Advanced Science and Engineering, Waseda University, 3-4-1 Okubo, Shinjuku-ku, Tokyo 169-8555, Japan; serendipity@asagi.waseda.jp (I.E.); furukawa@waseda.jp (Y.F.)

**Keywords:** femtosecond inverse Raman spectroscopy, conjugated polymer, bulk heterojunction, polaron

## Abstract

The initial charge separation process of conjugated polymers is one of the key factors for understanding their conductivity. The structure of photogenerated transients in conjugated polymers can be observed by resonance Raman spectroscopy in the near-IR region because they exhibit characteristic low-energy transitions. Here, we investigate the structure and dynamics of photogenerated transients in a regioregular poly(3-hexylthiophene) (P3HT):[6,6]-phenyl-C_61_-butyric acid methyl ester (PCBM) blend film, as well as in a pristine P3HT film, using femtosecond time-resolved resonance inverse Raman spectroscopy in the near-IR region. The transient inverse Raman spectrum of the pristine P3HT film at 50 ps suggests coexistence of neutral and charged excitations, whereas that of the P3HT:PCBM blend film at 50 ps suggests formation of positive polarons with a different structure from those in an FeCl_3_-doped P3HT film. Time-resolved near-IR inverse Raman spectra of the blend film clearly show the absence of charge separation between P3HT and PCBM within the instrument response time of our spectrometer, while they indicate two independent pathways of the polaron formation with time constants of 0.3 and 10 ps.

## 1. Introduction

The mechanism of conductivity in conjugated polymers has been an important problem in fundamental physical chemistry and material science since conductive polyacetylene was first synthesized [1]. Photoinduced conductivity of conjugated polymers [2,3] draws much attention for the understanding of photophysics in π-conjugated molecular systems as well as for the development of new materials functioning with solar energy.

Poly(3-alkylthiophene) (P3AT) is a fundamental conjugated polymer that exhibits high conductivity with chemical doping or with photoirradiation [4]. It has been widely accepted that the initial charge separation in P3AT is one of the key factors that determines the efficiency of the photoconductivity. Ultrafast time-resolved absorption and emission studies have been performed in the visible and near-IR regions to observe the dynamics of neutral and charged excitations formed in pristine P3AT films [5,6,7,8,9,10,11,12]. Pristine films of P3AT mainly show the self-localization dynamics of excitations initially created by a pump pulse in tens to hundreds of femtoseconds [7,8,10,11,12]. The excited-state dynamics of P3AT are significantly altered when P3AT is blended with fullerene derivatives in films [13,14,15,16,17,18,19,20], which is known to exhibit much higher power conversion efficiency than pristine films [21,22,23,24,25]. Time-resolved absorption studies [15,16,17,18,19] suggest that P3AT in blend films undergoes ultrafast charge separation with fullerene derivatives within their instrument response time, typically a few hundred femtoseconds, although the exact time constant has not been determined.

The structure of charge carriers, as well as their dynamics, is an important problem when it comes to understanding how charge carriers migrate in conjugated polymers. The structure of charge carriers in P3AT has been extensively studied using chemically or electrochemically doped films by IR and Raman spectroscopy. IR absorption of positive polarons in P3AT films are significantly enhanced [26,27,28] by electron–molecular vibration coupling [29,30]. Time-resolved IR vibrational spectroscopy has been performed for the direct observation of the structure of photogenerated charge carriers [27,31,32]. Although IR bands of charge carriers are clearly observed, their assignment has not been established yet because IR spectroscopy has no selectivity in observing charge carriers with different structures, such as positive polarons and bipolarons. The structure of charge carriers can be selectively observed when resonance Raman spectroscopy is used. Raman spectra of charge carriers have been reported for chemically or electrochemically doped P3AT films [33,34,35,36,37,38]. Time-resolved Raman spectroscopy has recently been reported for solutions of P3AT in isolated and aggregate forms [39,40] with the technique of femtosecond stimulated Raman spectroscopy (FSRS) [41,42]. FSRS is capable of investigating the ultrafast carrier formation dynamics in film samples as well as in solutions, as demonstrated by Hayes, Silva, and coworkers for a blend film of another conjugating polymer, PCDTBT, and a derivative of C_70_ [43]. It is difficult, however, to apply time-resolved Raman spectroscopy to films of conjugated polymers, because Raman spectroscopy usually requires a large photon flux and/or a long period of irradiation for the pump and probe pulses. Strong or long irradiation of film samples often causes irreversible photodamage in them.

In this study, we employ femtosecond time-resolved inverse Raman spectroscopy in the near-IR region in order to observe the structure of photogenerated charge carriers in a direct manner. In the inverse Raman measurement, the sample is irradiated with Raman pump and probe pulses with a frequency of ω_1_ and ω_2_, respectively, at the same time. Here ω_2_ is set in the anti-Stokes scattering frequency region with respect to ω_1_. A decrease of probe intensity is observed when the frequency difference, ω_2_–ω_1_, matches the frequency of a Raman active vibration. Femtosecond time-resolved inverse Raman spectroscopy can be performed with the same technique as FSRS, although they are different from each other in the optical process [44,45]. Significant suppression of photodamage can be expected for near-IR inverse Raman spectroscopy, because it can be performed with a small Raman pump frequency and much shorter exposure time. We present femtosecond time-resolved near-IR inverse Raman spectra of P3AT with hexyl side chains, P3HT, in a pristine film and in a film blended with [6,6]-phenyl-C_61_-butyric acid methyl ester (PCBM). The structure and dynamics of charge carriers in P3HT are discussed from the recorded spectra and their time evolution.

## 2. Results

### 2.1. Steady-State Near-IR Inverse Raman Spectra of Pristine and FeCl_3_-Doped P3HT Films

**Figure 1:**
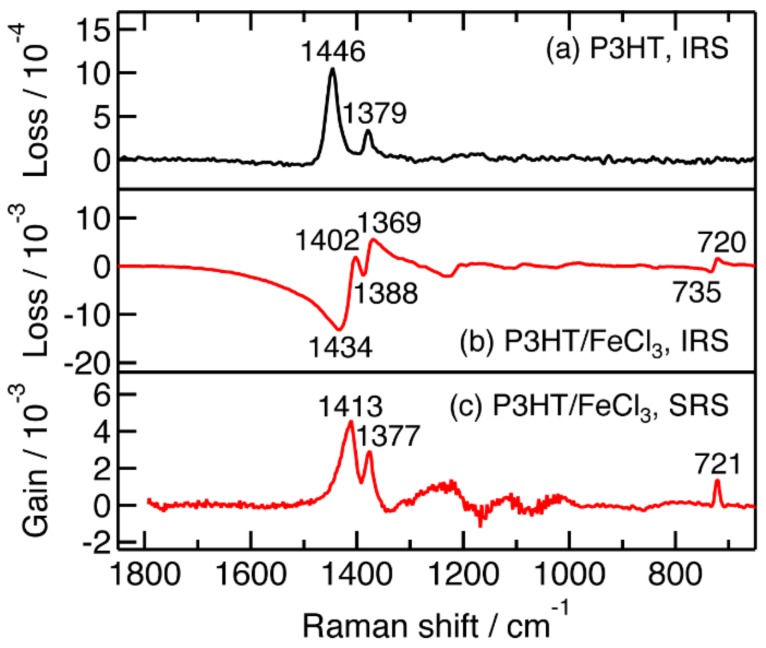
(**a**) The steady-state near-IR inverse Raman spectrum of a pristine P3HT film; (**b**) the steady-state near-IR inverse Raman spectrum of an FeCl_3_-doped P3HT film; (**c**) the steady-state stimulated Raman spectrum of an FeCl_3_-doped P3HT film. Broad bands between 1000 and 1300 cm^−1^ in (**c**) are artifacts originating from vibrational overtone absorption of water vapor in the air.

Steady-state inverse Raman spectra of pristine and FeCl_3_-doped P3HT films were recorded with the Raman pump and probe wavelength at 1190 nm and 900–1150 nm, respectively, for obtaining reference spectra of P3HT in the ground state and positively charged excited states. The results are shown in Figure 1. The pristine P3HT film shows a strong inverse Raman band at 1446 cm^−1^ and a weak band at 1379 cm^−1^ (Figure 1a). They are assigned to a C_α_=C_β_ symmetric stretch and a C_β_–C_β_ stretch vibrations of a thiophene ring [33,36]. Weak bands are observed at around 1200 and 720 cm^–1^ with intensity near the detection limit. The whole spectral pattern agrees well with the spontaneous Raman spectrum of a pristine P3HT film with excitation wavelength of 830 nm [36].

Inverse Raman bands of an FeCl_3_-doped P3HT film are observed with dispersive line shapes (Figure 1b) caused by the Raman pump and probe pulses in resonance with the near-IR transitions of the sample (Figure S1). The exact position of the band is not obtainable for a dispersive inverse Raman band unless the electronic resonance condition is fully determined [46]. We determined the exact positions of the bands of the FeCl_3_-doped P3HT film by observing its stimulated Raman scattering in the 1300–1550 nm region (Figure 1c). Positive peaks are clearly observed at 1413, 1377, and 721 cm^−1^, where the resonance inverse Raman bands are observed as well. The 1413 and 1377 cm^−1^ bands are assigned to ring C_α_C_β_ and C_β_C_β_ stretch vibrations of the positive polarons, in which the conjugate structure is significantly altered from neutral P3HT [33,36]. The 721 cm^–1^ band is assigned to a ring deformation vibration around the C–S–C bond [33]. The stimulated Raman spectrum of the FeCl_3_-doped P3HT film agrees well in position with their spontaneous Raman spectrum observed with the excitation wavelength of 780 nm [34] and 830 nm [36], although the width of the ring C_α_C_β_ stimulated Raman band appears smaller than the spontaneous one. The FeCl_3_-doped P3HT film does not show a Raman band characteristic of positive bipolarons in either the inverse or stimulated Raman spectrum. The negligible bipolaron generation is consistent with the previous studies performed by a group among the present authors [36,37].

### 2.2. Femtosecond Time-Resolved Near-IR Inverse Raman Spectra of Pristine P3HT and P3HT:PCBM Blend Films

**Figure 2:**
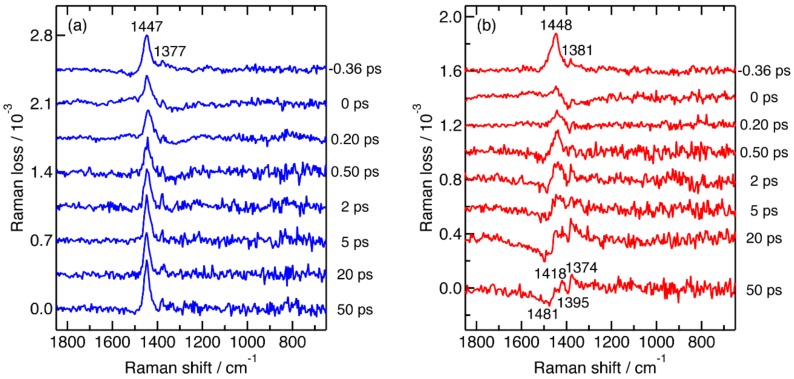
(**a**) Femtosecond time-resolved near-IR inverse Raman spectra of a pristine P3HT film; (**b**) femtosecond time-resolved near-IR inverse Raman spectra of a P3HT:PCBM blend film. The samples were photoexcited with energy density of 60 μJ cm^−2^ for the actinic pump pulse. Wavelengths of the actinic and Raman pump pulses was 480 and 1190 nm, respectively.

Femtosecond time-resolved near-IR inverse Raman spectra were recorded for pristine P3HT and P3HT:PCBM blend films with the actinic and Raman pump wavelengths at 480 and 1190 nm, respectively, for observing the structure of photogenerated transients as well as their dynamics. It has been established that two principal transients of P3HT, singlet excitons and positive polarons, exhibit broad absorption bands in the near-IR region (Figure S2). Therefore, resonance enhancement of inverse Raman bands of these transients can be expected under our experimental conditions. The obtained time-resolved inverse Raman spectra are shown in Figure 2. The spectra were recorded in a random order with respect to the time delay. Their baselines are fitted with 6th-order polynomial functions and subtracted (Figure S3). The spectra, after the baseline subtraction, contain the inverse Raman bands of P3HT in the ground state as well as those of photogenerated transients. The ground-state inverse Raman bands are not subtracted in Figure 2, because they appear as large ground-state depletion signals in the difference spectra and conceal the transient inverse Raman bands.

For a pristine P3HT film, the intensity of the ground-state inverse Raman bands slightly decreases from −0.36 to 0 ps due to the photoexcitation and almost fully recovers within a few picoseconds (Figure 2a). Inverse Raman bands of photogenerated transients, such as singlet excitons and positive polarons, are not observed in the time-resolved spectra. The intensity of their transient inverse Raman bands is below the detection limit of the spectrometer with the actinic pulse energy density of 60 μJ cm^−2^.

Significant time dependence is observed in femtosecond time-resolved near-IR inverse Raman spectra of a P3HT:PCBM blend film (Figure 2b). The intensity of the inverse Raman bands of P3HT in the ground state significantly decreases with the photoexcitation at 0 ps and then apparently recovers in part within 0.5 ps. Inverse Raman bands at around the C_α_C_β_ stretch region become dispersive after 2 ps from the photoexcitation. Because the shapes and peak positions of the dispersive bands agree reasonably well with those observed for the FeCl_3_-doped P3HT film (Figure 1b), they can be assigned to photogenerated positive polarons. The relative intensity of the positive peak at around 1418 cm^−1^ to that at around 1374 cm^−1^ decreases as time delay increases from 2 to 50 ps, indicating a slow change of transients in this time region.

### 2.3. Actinic Pump Energy Density Dependence of Femtosecond Time-Resolved Near-IR Inverse Raman Spectra

**Figure 3:**
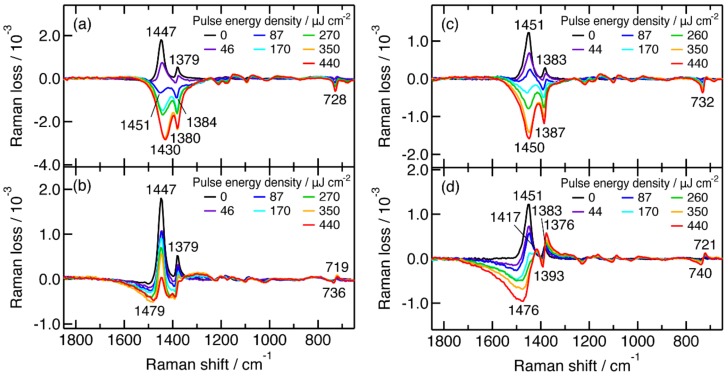
(**a**) Pulse energy density dependence of transient inverse Raman spectra of a pristine P3HT film at 0.20 ps; (**b**) a pristine P3HT film at 50 ps; (**c**) a P3HT:PCBM blend film at 0.20 ps; (**d**) a P3HT:PCBM blend film at 50 ps.

Transient near-IR inverse Raman spectra at 0.2 and 50 ps were recorded with increasing energy density of the actinic pump pulse from 0 to 4.4 × 10^2^ μJ cm^−2^, for observing the structure and dynamics of the photogenerated transients in detail. The results are shown in Figure 3. Here, baselines of the spectra are subtracted while inverse Raman bands of P3HT in the ground state are not subtracted.

For a pristine P3HT film, positive inverse Raman bands are observed at 1447 and 1379 cm^−1^ when the sample is not photoexcited at 480 nm (Figure 3a). The two bands turn to negative as the energy density of the actinic pump pulse increases. The negative bands can be assigned to singlet excitons in resonance with their near-IR transition, because they appear in the subpicosecond time scale and their shape is quite different from that of positive polarons (Figure 1b). The band intensity is proportional to energy density up to around 3.5 × 10^2^ μJ cm^−2^, although the correlation fluctuates because of uneven thickness of the samples. The linear intensity change shows that the number of the photogenerated singlet excitons is proportional to the number of actinic photons up to an energy density of around 3.5 × 10^2^ μJ cm^−2^. At 50 ps, a positive inverse Raman band is observed at the identical position with the ground-state band while the negative bands significantly decrease in intensity (Figure 3b). The positive band is assigned to the ground state partially recovered as a result of a prompt decay of singlet excitons. The detailed spectral shape of the negative bands is unclear because they are significantly overlapped with the ground state bands.

A P3HT:PCBM blend film shows an energy density dependence almost identical to that for the pristine P3HT film for their transient near-IR inverse spectrum at 0.2 ps (Figure 3c). At 50 ps, the intensity of the dispersive CC stretch bands of positive polarons increases with energy density (Figure 3d). The energy density dependence clearly shows that the center of the band is apparently down-shifted as the ground-state P3HT bands are more strongly depleted with high energy density. The positions of the minimum and maximum for the C_α_C_β_ stretch band are located at 1476 and 1417 cm^−1^, respectively, while they are estimated to be and 1434 and 1402 cm^−1^ for the FeCl_3_-doped P3HT film (Figure 1b).

## 3. Discussion

### 3.1. Structure of Photogenerated Singlet Excitons and Positive Polarons

Femtosecond time-resolved near-IR inverse Raman spectra of pristine P3HT and P3HT:PCBM blend films show substantial spectral changes as actinic pump energy density increases from 0 to 4.4 × 10^2^ μJ cm^−2^. Here, we discuss the structure of transients photogenerated in these films from the observed inverse Raman spectra with various actinic pump energy densities.

For the transient inverse Raman spectra of the pristine P3HT film at 0.2 ps, the peak position of the ring C_α_C_β_ stretch band, which is assigned to singlet excitons, appears to be down-shifted by 21 cm^−1^ as energy density increases from 80 to 3.5 × 10^2^ μJ cm^−2^ (Figure 3a). The intensity ratio of the ring C_α_C_β_ stretch band to the ring C_β_C_β_ stretch band increases by around a factor of 1.6 in the same range of energy densities. The downshift and the change in the intensity ratio have been reported for resonance Raman spectra of poly(3-decylthiophene) with increasing of the electrode potential [33]. These changes were explained by the increasing contribution of the quinoid structure by electrochemical oxidation, with the aid of the normal coordinate analysis in which the effects of charges are not considered. From the similarity of the spectral changes, we suggest that the photogenerated singlet excitons have a substantial contribution from the quinoid form in its resonance structure after strong irradiation of the actinic pump pulse. A similar trend is observed for the P3HT:PCBM blend film with a smaller downshift than that in the pristine P3HT film (Figure 3c). The smaller downshift can be interpreted as a smaller quinoid character of singlet excitons in the blend film than those in the pristine film. The quinoid character is perhaps suppressed when singlet excitons interact with PCBM molecules.

**Figure 4:**
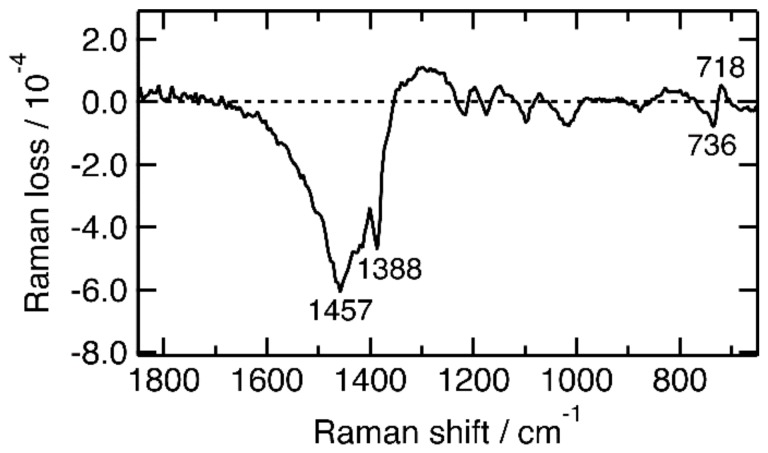
The difference inverse Raman spectrum of a pristine P3HT film at 50 ps between the spectra with energy density of 4.4 × 10^2^ and 0 μJ cm^−2^. The 0 μJ cm^−2^ spectrum was multiplied by 0.325 on the calculation of the difference spectrum.

The transient inverse Raman spectrum of the pristine P3HT film at 50 ps should contain information on structure of transients other than singlet excitons, because the shapes of the inverse Raman bands significantly differ from those at 0.2 ps. The inverse Raman bands at 50 ps, however, are not directly analyzable because of a serious overlap with those of the ground state. We tried to retrieve the transient inverse Raman bands by subtracting the 0 μJ cm^−2^ spectrum with a factor of 0.325 from the 4.4 × 10^2^ μJ cm^−2^ one. The result is shown in Figure 4. The difference spectrum shows a pattern similar to the spectrum of the singlet excitons (Figure 3a) in the 1800–1350 and 1250–750 cm^−1^ region. The spectrum is not simply assigned to singlet excitons, however, because clear differences are observed in the 1350–1250 and 750–700 cm^−1^ regions. In the difference spectrum, inverse Raman intensity is positive in 1350–1250 cm^−1^, and the ring C–S–C deformation band in 750–700 cm^−1^ has a dispersive shape. These features are not observed in the spectrum of the singlet excitons (Figure 3a) but observed in that of the positive polarons (Figure 1b). Thus, the difference spectrum suggests the coexistence of neutral and charged excitations. The coexistence of the two transients is consistent with the results of time-resolved studies in the visible to microwave region [5,6,9,10,47,48].

The transient inverse Raman spectrum of the P3HT:PCBM blend film at 50 ps shows a strong inverse Raman band with a dispersive line shape at around 1400 cm^–1^, which can safely be assigned to the C_α_C_β_ stretch band of positive polarons due to the similarity of the band shape to the inverse Raman bands of the FeCl_3_-doped P3HT film. The structure of positive polarons may differ from each other between the two samples, because the position of the C_α_C_β_ stretch bands disagree with each other. If we assume that the shape of the C_α_C_β_ stretch band does not significantly change due to the preparation method, we are able to estimate the center of the C_α_C_β_ stretch band of the positive polarons photogenerated in the P3HT:PCBM blend film from the following relation:

(1) $$\frac{\nu_{\max}{-\nu }_{c}}{\nu_{c}{-\nu }_{\min}}\;=\;\mathrm{const}.$$

Here *ν*_c_, *ν*_min_, and *ν*_max_ are the positions for the band center, the intensity minimum, and the intensity maximum of the C_α_C_β_ stretch band, respectively. In the case of the FeCl_3_-doped P3HT film, the positions of the minimum and maximum for the C_α_C_β_ stretch band are located at 1434 and 1402 cm^−1^, respectively, while the band center is estimated to be at 1413 cm^−1^ from the stimulated Raman spectrum of the same sample (Figure 1c). For the P3HT:PCBM blend film, the minimum and maximum are located at 1476 and 1417 cm^−1^, respectively. The band center is, therefore, determined to be at 1437 cm^−1^ for positive polarons photogenerated in the P3HT:PCBM blend film.

The center of the C_α_C_β_ stretch band of the positive polarons in the P3HT:PCBM blend film is up-shifted by 24 cm^−1^ from that in the FeCl_3_-doped P3HT film. The position of the C_α_C_β_ stretch band is down-shifted as doping time increases for in situ Raman measurements of a P3HT film in the FeCl_3_ vapor, which is most probably related to the effective delocalization length of charges or interaction between adjacent positive polarons [36]. Concentration of the photogenerated positive polarons will be much smaller than that of polarons generated by the FeCl_3_ doping. The downshift of 24 cm^−1^ can be explained by weaker interaction between positive polarons in the photoexcited P3HT:PCBM blend film than in the FeCl_3_-doped P3HT film.

### 3.2. Time Constants of Polaron Formation

When the P3HT:PCBM blend film is photoexcited with a large energy density, inverse Raman bands of singlet excitons and positive polarons become dominant in the transient inverse Raman spectra at 0.2 and 50 ps, respectively, while the ground state bands become negligible (Figure 3c,d). If we assume that the inverse Raman spectra at 0.2 and 50 ps with an actinic pump energy density of 4.4 × 10^2^ μJ cm^−2^ are assigned to singlet excitons and positive polarons, respectively, we can analyze the carrier formation dynamics from the time-resolved near-IR inverse Raman spectra of the P3HT:PCBM blend film. We fitted the time-resolved near-IR inverse Raman spectrum at each time delay with a linear combination of the three inverse Raman spectra representing the ground state, singlet excitons, and positive polarons. When multiple transients coexist in a sample, its inverse Raman intensity at a time delay *τ*, *I*(*τ*), is represented by the following expression:

(2)  I(τ) = ∑iIi(τ) ∝ −∑iIm χi,IRS(3)(τ) (i = 1,2,3,…)

Here *I_i_*(*τ*) and χ*_i_*_,IRS_^(3)^(*τ*) are the inverse Raman intensity and the 3rd-order nonlinear susceptibility of the *i-*th transient (*i* = 1, 2, 3,...), respectively. The χ*_i_*_,IRS_^(3)^(*τ*) value is proportional to the number density of the *i-*th transient at the time delay *τ*. The time-resolved inverse Raman spectra are satisfactorily reproduced when the Raman spectra of the three transients are used (Figure S4).

**Figure 5:**
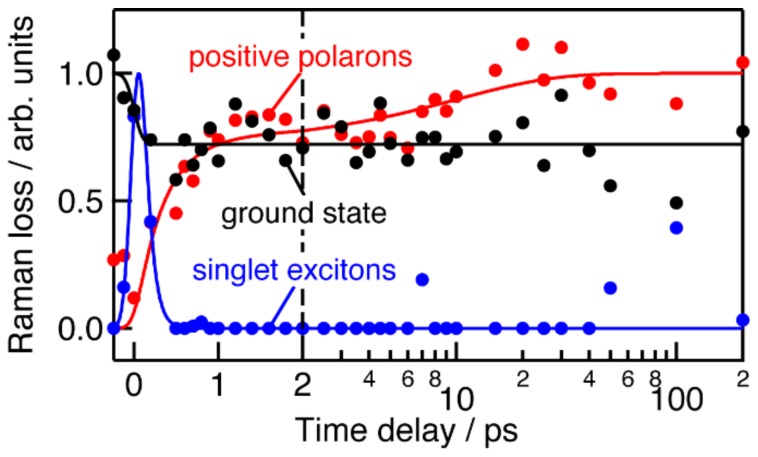
Time dependence of Raman loss for P3HT in the ground state, singlet exciton states, and positive polaron states in a P3HT:PCBM blend film. Energy density of the actinic pump pulse was 60 μJ cm^−2^. Filled circles represent the amplitude of the transients obtained by the least-squares fitting analysis of the time-resolved inverse Raman spectra with linear combinations of the spectra of the transients. Solid traces are the best fitted curves obtained by the least-squares fitting analysis with an error function, an exponential function and an offset, two exponential functions for the ground state, the singlet exciton states, and the positive polaron states, respectively.

The amplitudes of the three species were obtained and plotted against the time delay for obtaining their kinetics. The results are shown in Figure 5. Raman loss of the ground state decreases immediately after the sample is photoexcited with the energy density of 60 μJ cm^−2^. The rise of singlet excitons exactly matches the depletion of the ground state, indicating that the singlet excitons are formed at the moment of the photoexcitation. The singlet exciton bands fully decay within 1 ps while the ground state bands do not recover. The absence of the recovery strongly suggests that singlet excitons are almost quantitatively converted to other transients in the P3HT:PCBM film. The conversion efficiency is much higher than that of the pristine P3HT film, in which part of singlet excitons decay to the ground state within 50 ps (Figure 3b). The decay time constant of the singlet excitons is estimated to be 0.08 ps by the least-squares fitting analysis with an exponential function. The obtained time constant may be underestimated, however, because the singlet excitons provide much weaker inverse Raman bands than the ground state and the positive polarons (Figure 2b). The signal of the positive polarons rises substantially more slowly than the instrument response time of the spectrometer, 190 fs. The rise time constants are estimated to be 0.3 and 10 ps by the least-squares fitting analysis with two exponential functions. Neither of the time constants matches the decay time constant of the singlet excitons, indicating that part of the singlet excitons decay by singlet annihilation [16] and/or by formation of biexcitons.

The time constant of the initial charge separation has been estimated in P3HT:PCBM blend films by femtosecond time-resolved absorption [15,16,17,18,19] and fluorescence [49] spectroscopy. The reported time constants range from 500 fs to <100 fs, or within the instrument response time. Although the time constant obtained in this study, 0.3 ps, is located within the reported range, it does not agree with the result that the charge separation completes within the instrument response time [15,16,17,18,19]. It has been widely accepted that the charge separation dynamics are sensitive to the photoexcitation condition. The actinic pump density in the present study is 60 μJ cm^−2^, which is regarded as an extremely large value in recent studies. The high actinic pump density may slow down the charge separation, although Ohkita and his coworkers reported that the charge generation rate constant was independent of the actinic pump energy density up to 120 μJ cm^−2^ [17].

The two time constants suggest that positive polarons are generated from two kinds of transients in different manners. The following model has been suggested from time-resolved absorption studies [16,17,18,19]: (i) When singlet excitons are formed near the interface of the P3HT and PCBM domains, they can be promptly converted to positive polarons; (ii) when singlet excitons are generated in the bulk of the P3HT domain, they migrate to the interface of the P3HT and PCBM domains through carrier diffusion and undergo the charge separation with PCBM. The charge separation after the migration of the excitations provides the slow increase of the inverse Raman signal. If inverse Raman spectrum at 50 ps indicates that positive polarons are distributed predominantly around the interface of the P3HT and PCBM domains, the amplitude ratio of the two rise components indicates that around 70% of the positive polarons are generated directly from singlet excitons at the interface. If P3HT is photoexcited at the interface and in the bulk with an equal probability, 70% of P3HT in volume is regarded as forming the interface with PCBM. The large area of the effective interface estimated in this study is consistent with the high power conversion efficiency of P3HT:PCBM blend films.

At present, time-resolved inverse Raman spectroscopy is less sensitive than time-resolved absorption and fluorescence spectroscopy in investigation of the charge separation dynamics in conjugated polymer films. Time-resolved inverse Raman spectroscopy has an advantage, however, over time-resolved electronic spectroscopy, in that the inverse Raman signals of positive polarons can be easily distinguished from those of singlet excitons. The positive polarons show inverse Raman bands fully different from those of the singlet excitons in the band shape, as shown in Figure 3, owing to their resonance conditions. The band intensities of the positive polarons are almost comparable to those of the singlet excitons. These features enable us to retrieve the dynamics of positive polarons in a reliable manner.

## 4. Materials and Methods

Regioregular P3HT was purchased from Sigma-Aldrich (St. Louis, MI, USA). PCBM was purchased from Frontier Carbon. A P3HT solution was prepared in chlorobenzene with a concentration of P3HT (20 mg mL^−1^). A blend solution of P3HT and PCBM was prepared in chlorobenzene with a concentration of P3HT (20 mg mL^−1^) and PCBM (20 mg mL^−1^). A pristine P3HT film and a blend film were prepared from the solutions by the spin-coating method on a quartz substrate (20 × 20 mm, 1 mm thick) with absorbance of around 1 at 480 nm. A P3HT film for FeCl_3_ doping was prepared from a chlorobenzene solution (24 mg mL^−1^) by the spin-coating method on a glass substrate (20 × 20 mm, 1 mm thick) with absorbance of around 2.0. The film was soaked in 0.01 mol dm^–3^ FeCl_3_/acetonitrile solution for 40 s and then rinsed with pure acetonitrile for 50 s. Formation of positive polarons was confirmed by steady-state UV-visible spectroscopy (Figure S1) [36,50].

A lab-built femtosecond time-resolved near-IR multiplex stimulated/inverse Raman spectrometer was constructed [51,52,53] and used for recording absorption and inverse Raman spectra of transients photogenerated in pristine and blend films of P3HT. Details of the spectrometer have been described elsewhere [51,52]. Briefly, amplified output of a Ti:sapphire laser system (Vitesse/Legend Elite-HE, Coherent, Santa Clara, CA, USA, wavelength: 800 nm, pulse duration: 80 fs, repetition rate: 1 kHz) was divided into three parts for preparing the actinic pump (480 nm, 0.1–1.0 μJ), Raman pump (1190 nm, 1 μJ, ca. 3 cm^−1^), and probe (900–1550 nm) pulses. The transients were created with the actinic pump pulse and then their inverse Raman scattering was induced with the Raman pump and probe pulses after a time delay from the actinic pump pulse. Inverse Raman signals propagating along the Raman probe beam were detected by an InGaAs array detector (Symphony IGA, Horiba, Kyoto, Japan) equipped with a 32 cm spectrograph (iHR320, Horiba France, Longjumeau, France, spectral resolution: 5 cm^−1^). The Raman pump pulse was blocked in the time-resolved absorption measurement. Polarizations of the three pulses were set parallel with each other. The full width at half maximum of the instrument response function is estimated to be 190 fs for the time-resolved absorption and inverse Raman measurements of the film samples. All the spectra were recorded at 25 ± 1 °C under the aerated condition without covering the film samples by using another substrate, because the window in front of the sample film provided strong artifact signals. For avoiding accumulation of photodamage at the focus point, the samples were translationally moved every 40 s during the time-resolved near-IR inverse Raman measurements. The stationary inverse Raman spectrum with the pump irradiation was unchanged from that recorded without the pump irradiation (Figure S5). The effects of oxygen and strong photoirradiation were negligible on the recorded time-resolved near-IR inverse Raman spectra.

## 5. Conclusions

In this study, we have performed femtosecond time-resolved near-IR inverse Raman spectroscopy for investigating the structure and dynamics of photogenerated transients in pristine P3HT and P3HT:PCBM blend films. Time-resolved near-IR inverse Raman spectra of a pristine P3HT film indicate decrease of transients within 50 ps and the coexistence of neutral and charged excitations at 50 ps or later. A P3HT:PCBM blend film shows signals of positive polarons in addition to the three transients observed in the pristine P3HT film. The structure of the photogenerated positive polarons is similar to that of positive polarons in an FeCl_3_-doped P3HT film, but not identical to it, because the photogenerated positive polarons can interact only weakly with each other due to their significantly low concentration.

The time-resolved near-IR inverse Raman spectra reveal the initial charge separation dynamics in the P3HT:PCBM blend film. Positive polarons are generated with a time constant of 0.3 ps when the initially created singlet excitons are located nearby PCBM. They further increase with a time constant of 10 ps by slow charge separation with PCBM after the migration of excitations in the bulk of P3HT. Time-resolved inverse Raman spectra of the P3HT:PCBM blend film provide information on the volume of P3HT forming the interface with PCBM. The volume is estimated to be around 70% for the P3HT:PCBM blend film with the mass ratio of P3HT to PCBM of 1:1. Femtosecond time-resolved inverse Raman spectroscopy is an effective tool for estimating the efficiency of the carrier generation through direct observation of the polaron formation at the interface.

## Supplementary Materials

The following are available online. Figure S1: Steady-state absorption spectra; Figure S2: Femtosecond time-resolved near-IR absorption spectra; Figure S3: Fitting analysis of baselines; Figure S4: Least-squares fitting analysis of time-resolved inverse Raman spectra; Figure S5: Effects of actinic pump irradiation on film samples.
