# Supplementary material for: Direct Observation of Structure and Dynamics of Photogenerated Charge Carriers in Poly(3-hexylthiophene) Films by Femtosecond Time-Resolved Near-IR Inverse Raman Spectroscopy
Source: Molecules. 2019 Jan 25;24(3):431. doi: 10.3390/molecules24030431 (PMC6384712; doi:10.3390/molecules24030431)
Supplement: Supplementary file 1 [file molecules-24-00431-s001.pdf]

Supporting Information

Direct Observation of Structure and Dynamics of Photogenerated Charge Carriers in Poly(3-hexylthiophene) Films by Femtosecond Time-Resolved Near-IR Inverse Raman Spectroscopy

Tomohisa Takaya [1,*], Ippei Enokida [2], Yukio Furukawa [2], and Koichi Iwata [1,*]

[1] Department of Chemistry, Faculty of Science, Gakushuin University, 1-5-1 Mejiro, Toshima-ku, Tokyo 171-8588, Japan
[2] Department of Advanced Science and Engineering, Waseda University, 3-4-1 Okubo, Shinjuku-ku, Tokyo 169-8555, Japan

* Correspondence: tomohisa.takaya@gakushuin.ac.jp; Tel.: +81-3-5904-9386 (T.T.); koichi.iwata@gakushuin.ac.jp; Tel.: +81-3-5904-9385 (K.I.)

Steady-state absorption spectra of film samples

Steady-state absorption spectra were recorded for the same samples used in the time-resolved absorption and inverse Raman measurements. The absorption spectrum of a pristine PCBM film was recorded for estimating relative absorbance of P3HT in a P3HT:PCBM blend film. The pristine PCBM film was prepared by the spin-coating method. PCBM was dissolved in chloroform (20 mg mL^{-1}) and spin-coated on a quartz substrate (20 x 20 mm, 1 mm thick) with absorbance of around 0.6 at 480 nm. The obtained spectra are shown in Figure S1. The absorption spectrum of the P3HT:PCBM blend film is reproduced when the absorption spectra of the pristine films of P3HT and PCBM are scaled to absorbance of 0.8 and 0.2 at 480 nm, respectively, and added. Around 80% of the photons in an actinic pump pulse are expected to be absorbed by P3HT in the blend film.

The absorption spectrum of an $FeCl_3$-doped P3HT film shows two absorption bands at around 800 nm and in the 1200–1600 nm region. They are assigned to positive polarons [1]. Positive bipolarons are negligible in the film used in this study.

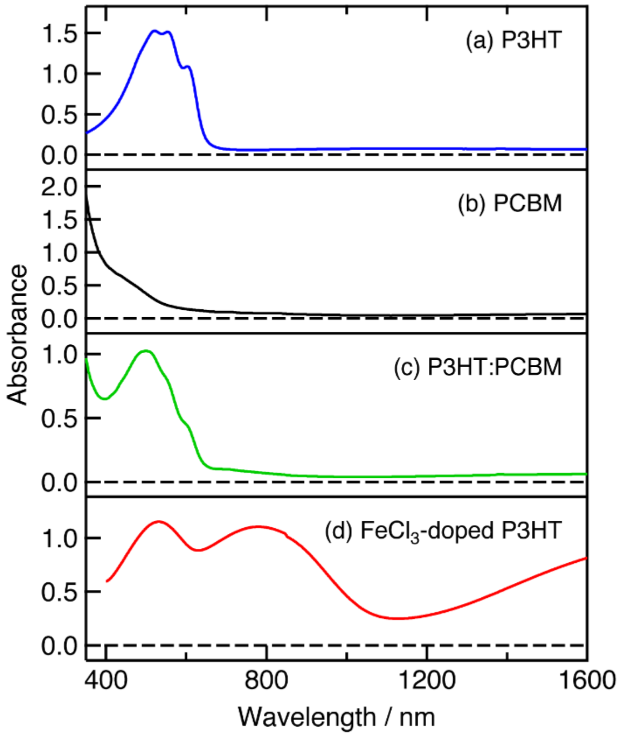

Figure S1. (**a**) Steady-state absorption spectra of a pristine P3HT film; (**b**) a pristine PCBM film; (**c**) a P3HT:PCBM blend film; (**d**) an $FeCl_3$-doped P3HT film.

Femtosecond time-resolved near-IR absorption spectra of film samples

The initial charge separation process in a pristine P3HT film was observed by femtosecond time-resolved near-IR absorption spectroscopy in the 900–1400 nm region. The sample film was photoexcited at 480 nm with energy density of 60 μJ cm^{-2}. The results are shown in Figure S2a. An excited-state absorption band of P3HT in the pristine film appears immediately after the photoexcitation, with a peak at 1200 nm. The peak position apparently shifts by 100 nm between 0.2 and 2 ps while a subpeak rises at around 1100 nm. The absorption at 1300 nm decays and almost disappears in 50 ps, whereas the subpeak at 1100 nm remains at 50 ps. From reported time-resolved absorption spectra [2], we assigned the initial absorption bands at 0 and 50 ps to singlet excitons and positive polarons. We think, however, the transient at 50 ps has a character of singlet excitons as well as that of positive polarons (See Section 3.1 in the main text).

Time-resolved near-IR absorption spectra were recorded for a P3HT:PCBM blend film with the same actinic pump wavelength and energy density that were used for the measurement of the pristine P3HT film. The actinic pump pulse mainly photoexcited P3HT in the blend film (Figure S1). The results are shown in Figure S2b. A broad absorption band appears at 1050 nm immediately after the photoexcitation of the P3HT:PCBM blend film, which can be assigned to singlet excitons. The spectrum at 0.5 ps show a small and narrow band at around 1030 nm and a broad feature covering the entire wavelength region from 900 to 1400 nm. The broad feature decays significantly within 5 ps while the intensity of the narrow band is almost unchanged. The spectrum at 50 ps reveals that the peak at 1030 nm is a part of absorption from 1100 nm to the visible region. The absorption band at 50 ps can be assigned to positive polarons in the blend film from similarity to reported spectra [3].

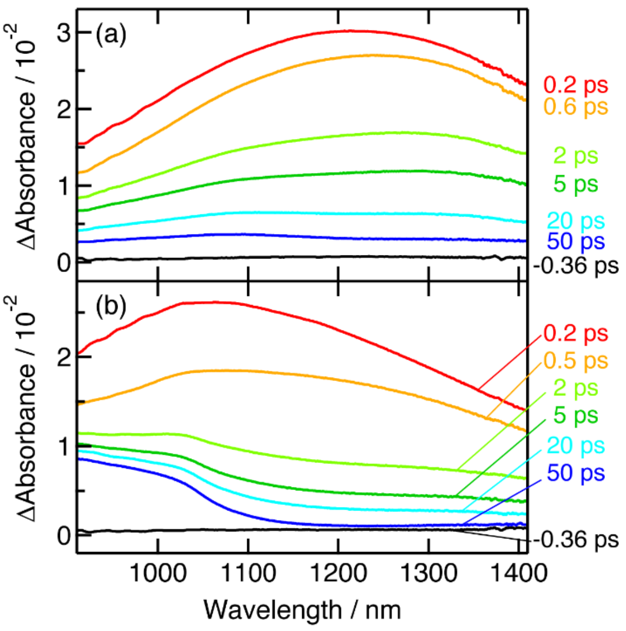

Figure S2. (**a**) Femtosecond time-resolved near-IR absorption spectra of a pristine P3HT film; (**b**) Femtosecond time-resolved near-IR absorption spectra of a P3HT:PCBM blend film. The samples were photoexcited with an energy density of 60 μJ cm^{-2} for the actinic pump pulse. The actinic pump wavelength was 480 nm.

Baseline correction for time-resolved near-IR inverse Raman spectra

Inverse Raman bands of samples are observed on a complex baseline provided by nonlinear optical processes other than inverse Raman scattering, such as pumping of transients to higher excited states by a Raman pump pulse and multiphoton absorption [4]. We fitted the baseline of the inverse Raman spectrum with a polynomial function and subtracted it from the raw spectrum. Figure S3 shows the results of the fitting for time-resolved inverse Raman spectra of pristine P3HT and P3HT:PCBM blend films. The baseline correction was applied to steady-state inverse Raman spectra of film samples as well (Figure 3 in the main text).

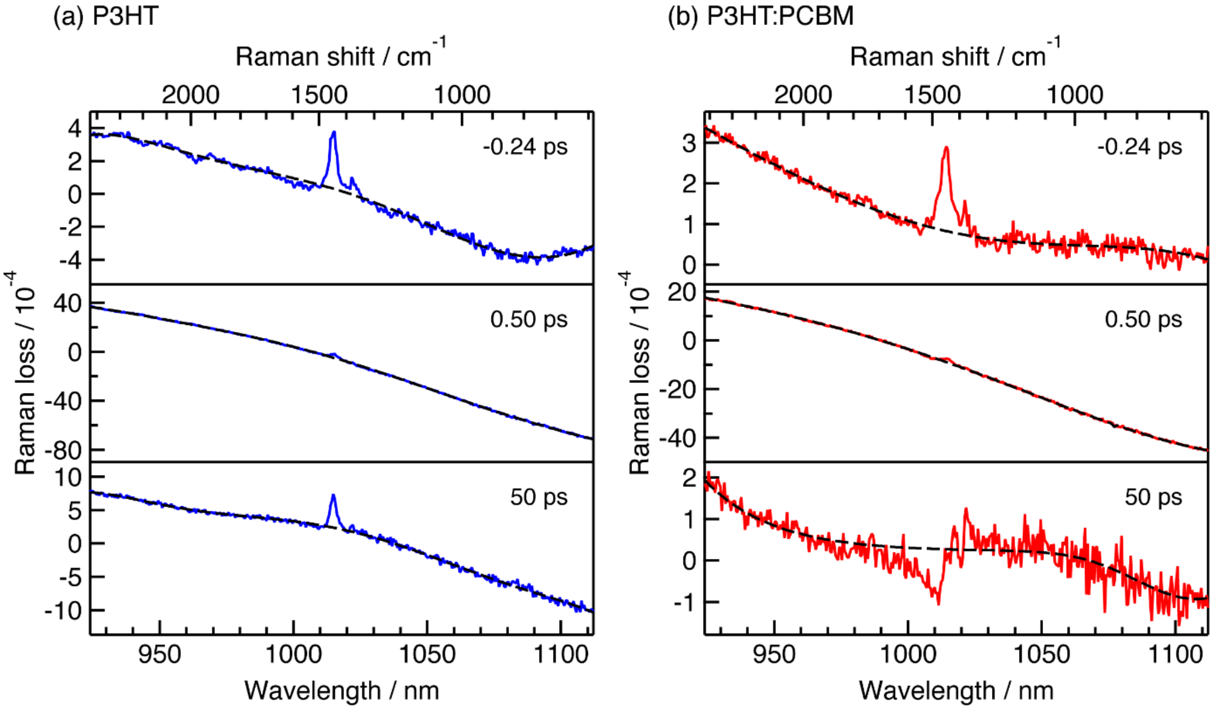

Figure S3. (**a**) Fitting analysis of baselines with polynomial functions for time-resolved inverse Raman spectra of a pristine P3HT film; (**b**) a P3HT:PCBM blend film. The blue and red traces are the raw spectra for P3HT and P3HT:PCBM blend films, respectively. The fitted curves are shown with broken traces.

Least-squares fitting analysis using linear combination of transient spectra

As shown in Equation 2 the main text, the inverse Raman intensity is linearly correlated with the third-order nonlinear susceptibility. An inverse Raman spectrum of a sample with multiple components is, therefore, represented by a linear combination of the component spectra.

We assumed that three species, the ground state (gs), singlet excitons (se), and positive polarons (pp), are principally involved in time-resolved inverse Raman spectra of a P3HT:PCBM film and analyzed the spectrum at each time delay using the linear combination of the three spectra,

$$I = c_{\mathrm{gs}}I_{\mathrm{gs}} + c_{\mathrm{se}}I_{\mathrm{se}} + c_{\mathrm{pp}}I_{\mathrm{pp}}. \tag{S1}$$

Here c and I are the relative amplitude and inverse Raman intensity, respectively. The relative intensity of the three spectra, I_{gs}, I_{se}, and I_{pp}, is not necessary to be normalized because absolute third-order nonlinear susceptibility of the three species is unknown. The results of the fitting analysis are shown in Figure S4 for the time-resolved inverse Raman spectra at 0.50, 5, and 50 ps from the photoexcitation. The spectra at the three time delays are well reproduced by the linear combinations of the spectra of the three transients, the ground state, singlet excitons, and positive polarons. The plot of the c_{gs}, c_{se}, and c_{pp} values against the time delay is shown in Figure 5 in the main text.

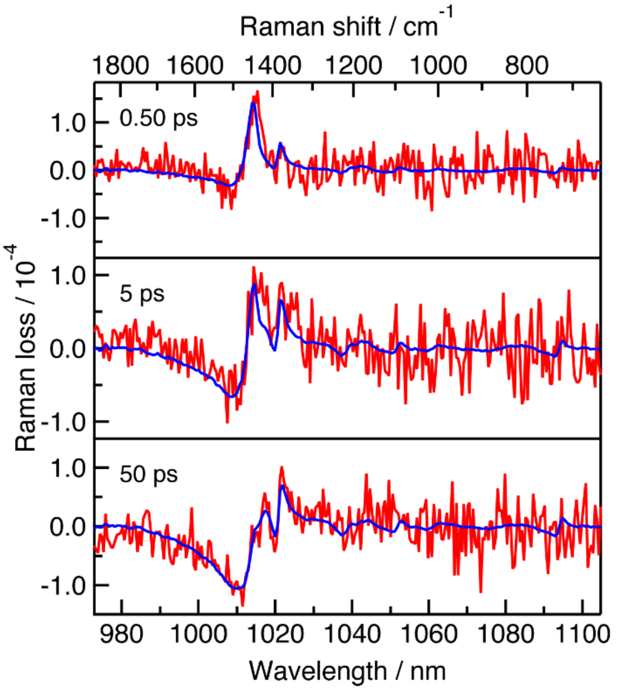

Figure S4. Least-squares fitting analysis of time-resolved inverse Raman spectra of a P3HT:PCBM blend film at 0.50, 5, and 50 ps using Equation S1. The red and blue traces represent the observed spectra and best-fitted curves, respectively.

Effects of actinic pump irradiation on film samples

It has been known that a large photon flux and/or long period of actinic pump irradiation often induces photodamage in film samples. The accumulation of photodamage can be avoided if the actinic pump energy density is sufficiently decreased and films are set in nitrogen or vacuum. In our study, however, the samples are set in the air for avoiding artifact signals from a window in front of them.

Since the samples are exposed to oxygen in the air, we carefully confirmed that the photodamage is not accumulated on them by recording their stationary inverse Raman spectra in the absence of the actinic pump pulse before recording the spectra under the actinic pump irradiation. The results are shown in Figure S5. The figure clearly shows that the intensities and positions of the inverse Raman bands at around 1400 cm^{-1} are unchanged within 40 s of the actinic pump irradiation, although baselines of the spectra are slightly fluctuated due to instability of the probe spectrum. The results indicate that the influences of the strong actinic pump irradiation do not appear when the samples are translationally moved every 40 s.

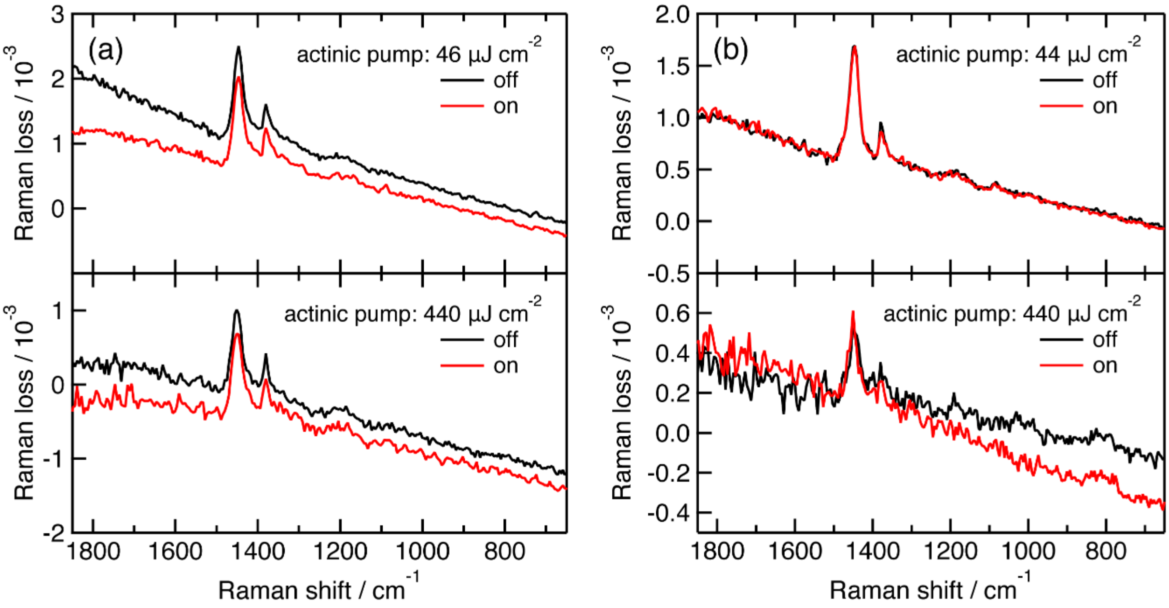

Figure S5. (**a**) Stationary inverse Raman spectra of a pristine P3HT film with actinic pump irradiation (red trace) and without irradiation (black trace) at two different actinic pump energy densities. (**b**) Stationary inverse Raman spectra of a P3HT:PCBM blend film with actinic pump irradiation (red trace) and without irradiation (black trace). The actinic pump pulse arrives at the samples at least 1 ps before the Raman pump and probe pulses arrive. The exposure time is 40 s.

References

1. Yamamoto, J.; Furukawa, Y. Electronic and Vibrational Spectra of Positive Polarons and Bipolarons in Regioregular Poly(3-hexylthiophene) Doped with Ferric Chloride. *J. Phys. Chem. B* **2015**, *119*, 4788–4794.
2. Guo. J.; Ohkita, H.; Benten, H.; Ito, S. Near-IR Femtosecond Transient Absorption Spectroscopy of Ultrafast Polaron and Triplet Exciton Formation in Polythiophene Films with Different Regioregularities. *J. Am. Chem. Soc.* **2009**, *131*, 16869–16880.
3. Guo. J.; Ohkita, H.; Benten, H.; Ito, S. Charge Generation and Recombination Dynamics in Poly(3-hexylthiophene)/Fullerene Blend Films with Different Regioregularities and Morphologies. *J. Am. Chem. Soc.* **2010**, *132*, 6154–6164.
4. Takaya, T.; Iwata, K. Relaxation Mechanism of β-carotene from S_2 ($1B_u^+$) State to S_1 ($2A_g^-$) State: Femtosecond Time-Resolved Near-IR Absorption and Stimulated Resonance Raman Studies in 900–1550 nm Region. *J. Phys. Chem. A* **2014**, *118*, 4071–4078.
